# Supplementary material for: Multi‐dataset identification of innovative feature genes and molecular mechanisms in keratoconus
Source: J Cell Mol Med. 2024 Sep 19;28(18):e70079. doi: 10.1111/jcmm.70079 (PMC11412914; doi:10.1111/jcmm.70079)
Supplement: Supplementary file 2 — Material S2. [file JCMM-28-e70079-s002.zip › 7.GSEA analysis/6.SNORA74B/KEGG_HUNTINGTONS_DISEASE.html]

Details for gene set KEGG\_HUNTINGTONS\_DISEASE[GSEA]

|  || Dataset | SNORA74B.group.cls#h\_versus\_l |
| Phenotype | group.cls#h\_versus\_l |
| Upregulated in class | l |
| GeneSet | KEGG\_HUNTINGTONS\_DISEASE |
| Enrichment Score (ES) | -0.25597337 |
| Normalized Enrichment Score (NES) | -1.4485167 |
| Nominal p-value | 0.08219178 |
| FDR q-value | 0.3237355 |
| FWER p-Value | 0.851 |
Table: GSEA Results Summary

  

Fig 1: Enrichment plot: KEGG\_HUNTINGTONS\_DISEASE      
 Profile of the Running ES Score & Positions of GeneSet Members on the Rank Ordered List

  

| SYMBOL | TITLE | RANK IN GENE LIST | RANK METRIC SCORE | RUNNING ES | CORE ENRICHMENT || 1 | CLTC | na | 2065 | 0.366 | -0.0294 | No |
| 2 | SLC25A31 | na | 2594 | 0.326 | -0.0175 | No |
| 3 | GRM5 | na | 3432 | 0.281 | -0.0184 | No |
| 4 | TBPL2 | na | 5327 | 0.214 | -0.0554 | No |
| 5 | HTT | na | 7868 | 0.139 | -0.1173 | No |
| 6 | APAF1 | na | 8465 | 0.125 | -0.1242 | No |
| 7 | CASP8 | na | 9153 | 0.110 | -0.1349 | No |
| 8 | PLCB3 | na | 9334 | 0.106 | -0.1313 | No |
| 9 | PLCB2 | na | 9573 | 0.101 | -0.1298 | No |
| 10 | CASP3 | na | 9789 | 0.097 | -0.1280 | No |
| 11 | COX6B2 | na | 9923 | 0.094 | -0.1240 | No |
| 12 | CREBBP | na | 10525 | 0.083 | -0.1345 | No |
| 13 | AP2A2 | na | 10586 | 0.082 | -0.1294 | No |
| 14 | UCP1 | na | 11536 | 0.066 | -0.1514 | No |
| 15 | SP1 | na | 11587 | 0.066 | -0.1474 | No |
| 16 | GNAQ | na | 11760 | 0.063 | -0.1471 | No |
| 17 | NDUFB5 | na | 11783 | 0.063 | -0.1425 | No |
| 18 | POLR2H | na | 12060 | 0.059 | -0.1456 | No |
| 19 | NDUFA10 | na | 12175 | 0.057 | -0.1441 | No |
| 20 | NDUFS1 | na | 12266 | 0.056 | -0.1420 | No |
| 21 | POLR2J2 | na | 12274 | 0.056 | -0.1375 | No |
| 22 | REST | na | 12595 | 0.052 | -0.1425 | No |
| 23 | DNAI2 | na | 12932 | 0.048 | -0.1482 | No |
| 24 | DNAI1 | na | 13229 | 0.044 | -0.1531 | No |
| 25 | COX6A2 | na | 13423 | 0.042 | -0.1552 | No |
| 26 | COX4I2 | na | 13609 | 0.040 | -0.1571 | No |
| 27 | NDUFS6 | na | 13670 | 0.040 | -0.1556 | No |
| 28 | POLR2C | na | 14386 | 0.031 | -0.1736 | No |
| 29 | RCOR1 | na | 14389 | 0.031 | -0.1711 | No |
| 30 | NDUFV3 | na | 14510 | 0.030 | -0.1721 | No |
| 31 | NDUFV1 | na | 14579 | 0.029 | -0.1716 | No |
| 32 | COX7A2L | na | 14598 | 0.029 | -0.1697 | No |
| 33 | NDUFB1 | na | 14612 | 0.029 | -0.1677 | No |
| 34 | UQCR11 | na | 14688 | 0.028 | -0.1675 | No |
| 35 | NDUFA1 | na | 14854 | 0.026 | -0.1702 | No |
| 36 | NDUFC2 | na | 15136 | 0.023 | -0.1764 | No |
| 37 | NDUFS5 | na | 15558 | 0.019 | -0.1870 | No |
| 38 | COX5B | na | 15566 | 0.019 | -0.1856 | No |
| 39 | POLR2A | na | 15608 | 0.018 | -0.1853 | No |
| 40 | COX6B1 | na | 15616 | 0.018 | -0.1839 | No |
| 41 | COX7B | na | 15639 | 0.018 | -0.1831 | No |
| 42 | CREB3 | na | 15657 | 0.018 | -0.1821 | No |
| 43 | NDUFS8 | na | 15671 | 0.018 | -0.1809 | No |
| 44 | NDUFAB1 | na | 15832 | 0.016 | -0.1842 | No |
| 45 | CREB3L1 | na | 15845 | 0.016 | -0.1832 | No |
| 46 | SDHC | na | 15907 | 0.016 | -0.1837 | No |
| 47 | NDUFC1 | na | 15955 | 0.015 | -0.1838 | No |
| 48 | GPX1 | na | 16065 | 0.014 | -0.1858 | No |
| 49 | NDUFB8 | na | 16113 | 0.014 | -0.1860 | No |
| 50 | COX6C | na | 16123 | 0.013 | -0.1852 | No |
| 51 | CLTA | na | 16174 | 0.013 | -0.1856 | No |
| 52 | POLR2J3 | na | 16190 | 0.013 | -0.1849 | No |
| 53 | NDUFB10 | na | 16396 | 0.010 | -0.1900 | No |
| 54 | COX7A2 | na | 16504 | 0.009 | -0.1923 | No |
| 55 | NDUFB3 | na | 16657 | 0.008 | -0.1960 | No |
| 56 | AP2M1 | na | 16749 | 0.007 | -0.1981 | No |
| 57 | NDUFS4 | na | 16762 | 0.007 | -0.1979 | No |
| 58 | PLCB4 | na | 16838 | 0.006 | -0.1995 | No |
| 59 | NDUFB9 | na | 16921 | 0.005 | -0.2015 | No |
| 60 | COX8A | na | 16941 | 0.005 | -0.2016 | No |
| 61 | NDUFS3 | na | 16944 | 0.005 | -0.2012 | No |
| 62 | HDAC1 | na | 16993 | 0.005 | -0.2022 | No |
| 63 | POLR2I | na | 17022 | 0.005 | -0.2026 | No |
| 64 | DCTN2 | na | 17094 | 0.004 | -0.2044 | No |
| 65 | UQCRQ | na | 17096 | 0.004 | -0.2041 | No |
| 66 | POLR2J | na | 17188 | 0.003 | -0.2065 | No |
| 67 | UQCRC1 | na | 17305 | 0.001 | -0.2097 | No |
| 68 | NDUFB4 | na | 17386 | 0.001 | -0.2120 | No |
| 69 | NDUFA8 | na | 17501 | -0.001 | -0.2152 | No |
| 70 | COX7C | na | 17567 | -0.001 | -0.2170 | No |
| 71 | UQCRC2 | na | 17583 | -0.002 | -0.2173 | No |
| 72 | NDUFB7 | na | 17800 | -0.004 | -0.2232 | No |
| 73 | SLC25A5 | na | 17839 | -0.004 | -0.2240 | No |
| 74 | NDUFS7 | na | 17903 | -0.005 | -0.2254 | No |
| 75 | NDUFA7 | na | 17983 | -0.006 | -0.2272 | No |
| 76 | NDUFB2 | na | 18027 | -0.006 | -0.2280 | No |
| 77 | NDUFA5 | na | 18127 | -0.007 | -0.2302 | No |
| 78 | NDUFA3 | na | 18279 | -0.009 | -0.2338 | No |
| 79 | VDAC2 | na | 18356 | -0.010 | -0.2352 | No |
| 80 | CREB3L3 | na | 18368 | -0.010 | -0.2346 | No |
| 81 | AP2S1 | na | 18845 | -0.015 | -0.2471 | No |
| 82 | COX6A1 | na | 18860 | -0.015 | -0.2462 | No |
| 83 | PPID | na | 18891 | -0.016 | -0.2458 | No |
| 84 | SDHD | na | 18918 | -0.016 | -0.2452 | No |
| 85 | BAX | na | 18953 | -0.016 | -0.2448 | No |
| 86 | COX5A | na | 19063 | -0.018 | -0.2465 | No |
| 87 | POLR2G | na | 19301 | -0.021 | -0.2516 | No |
| 88 | UQCR10 | na | 19319 | -0.021 | -0.2504 | No |
| 89 | SLC25A6 | na | 19514 | -0.023 | -0.2541 | Yes |
| 90 | VDAC3 | na | 19531 | -0.023 | -0.2526 | Yes |
| 91 | TP53 | na | 19572 | -0.024 | -0.2518 | Yes |
| 92 | NDUFA9 | na | 19672 | -0.025 | -0.2526 | Yes |
| 93 | UQCRFS1 | na | 19694 | -0.025 | -0.2511 | Yes |
| 94 | NDUFA4 | na | 19698 | -0.025 | -0.2491 | Yes |
| 95 | DNAH1 | na | 19756 | -0.026 | -0.2486 | Yes |
| 96 | POLR2L | na | 19908 | -0.027 | -0.2506 | Yes |
| 97 | NDUFA2 | na | 19988 | -0.028 | -0.2506 | Yes |
| 98 | NDUFA6 | na | 20118 | -0.030 | -0.2519 | Yes |
| 99 | POLR2E | na | 20158 | -0.030 | -0.2505 | Yes |
| 100 | DNAL4 | na | 20336 | -0.032 | -0.2529 | Yes |
| 101 | DCTN1 | na | 20370 | -0.032 | -0.2512 | Yes |
| 102 | UQCRH | na | 20531 | -0.034 | -0.2530 | Yes |
| 103 | COX4I1 | na | 20535 | -0.034 | -0.2502 | Yes |
| 104 | NDUFV2 | na | 20612 | -0.035 | -0.2495 | Yes |
| 105 | SDHA | na | 20629 | -0.035 | -0.2470 | Yes |
| 106 | POLR2D | na | 20657 | -0.036 | -0.2448 | Yes |
| 107 | DCTN4 | na | 20846 | -0.038 | -0.2471 | Yes |
| 108 | TFAM | na | 20892 | -0.039 | -0.2452 | Yes |
| 109 | TBP | na | 21006 | -0.040 | -0.2451 | Yes |
| 110 | UQCRB | na | 21097 | -0.041 | -0.2443 | Yes |
| 111 | SOD1 | na | 21117 | -0.041 | -0.2414 | Yes |
| 112 | CREB3L4 | na | 21237 | -0.043 | -0.2413 | Yes |
| 113 | POLR2F | na | 21583 | -0.047 | -0.2474 | Yes |
| 114 | TBPL1 | na | 21624 | -0.047 | -0.2446 | Yes |
| 115 | CASP9 | na | 21843 | -0.050 | -0.2468 | Yes |
| 116 | POLR2B | na | 21915 | -0.051 | -0.2446 | Yes |
| 117 | HDAC2 | na | 22146 | -0.053 | -0.2469 | Yes |
| 118 | CREB3L2 | na | 22159 | -0.054 | -0.2427 | Yes |
| 119 | NDUFB6 | na | 22187 | -0.054 | -0.2391 | Yes |
| 120 | CYCS | na | 22291 | -0.055 | -0.2374 | Yes |
| 121 | AP2B1 | na | 22298 | -0.055 | -0.2330 | Yes |
| 122 | TAF4 | na | 22641 | -0.060 | -0.2379 | Yes |
| 123 | NDUFS2 | na | 22695 | -0.061 | -0.2344 | Yes |
| 124 | CYC1 | na | 23261 | -0.069 | -0.2450 | Yes |
| 125 | VDAC1 | na | 23601 | -0.075 | -0.2486 | Yes |
| 126 | SLC25A4 | na | 23611 | -0.075 | -0.2426 | Yes |
| 127 | COX7A1 | na | 23663 | -0.076 | -0.2378 | Yes |
| 128 | SDHB | na | 23674 | -0.076 | -0.2318 | Yes |
| 129 | CLTB | na | 23698 | -0.076 | -0.2262 | Yes |
| 130 | ITPR1 | na | 23722 | -0.077 | -0.2205 | Yes |
| 131 | NDUFA4L2 | na | 24044 | -0.081 | -0.2230 | Yes |
| 132 | GRIN2B | na | 24135 | -0.083 | -0.2187 | Yes |
| 133 | SOD2 | na | 24371 | -0.087 | -0.2183 | Yes |
| 134 | SIN3A | na | 24592 | -0.091 | -0.2171 | Yes |
| 135 | POLR2K | na | 24648 | -0.092 | -0.2110 | Yes |
| 136 | GRIN1 | na | 25109 | -0.100 | -0.2160 | Yes |
| 137 | EP300 | na | 25385 | -0.105 | -0.2153 | Yes |
| 138 | CREB1 | na | 25963 | -0.116 | -0.2223 | Yes |
| 139 | AP2A1 | na | 26356 | -0.124 | -0.2233 | Yes |
| 140 | DNALI1 | na | 26563 | -0.129 | -0.2186 | Yes |
| 141 | HIP1 | na | 26756 | -0.134 | -0.2130 | Yes |
| 142 | HAP1 | na | 26892 | -0.137 | -0.2055 | Yes |
| 143 | BBC3 | na | 27165 | -0.143 | -0.2015 | Yes |
| 144 | PPARGC1A | na | 27305 | -0.147 | -0.1933 | Yes |
| 145 | DNAL1 | na | 28016 | -0.165 | -0.2001 | Yes |
| 146 | DLG4 | na | 28732 | -0.187 | -0.2053 | Yes |
| 147 | BDNF | na | 28985 | -0.194 | -0.1964 | Yes |
| 148 | COX7B2 | na | 28987 | -0.195 | -0.1803 | Yes |
| 149 | TAF4B | na | 29261 | -0.203 | -0.1713 | Yes |
| 150 | NRF1 | na | 30925 | -0.264 | -0.1975 | Yes |
| 151 | CLTCL1 | na | 30955 | -0.265 | -0.1763 | Yes |
| 152 | PPARG | na | 31596 | -0.300 | -0.1699 | Yes |
| 153 | DNAH2 | na | 32575 | -0.359 | -0.1684 | Yes |
| 154 | PLCB1 | na | 32854 | -0.381 | -0.1448 | Yes |
| 155 | COX8C | na | 33258 | -0.420 | -0.1216 | Yes |
| 156 | IFT57 | na | 33948 | -0.523 | -0.0981 | Yes |
| 157 | TGM2 | na | 34358 | -0.636 | -0.0571 | Yes |
| 158 | CREB5 | na | 34618 | -0.816 | 0.0032 | Yes |
Table: GSEA details [plain text format]

  

Fig 2: KEGG\_HUNTINGTONS\_DISEASE      
 Blue-Pink O' Gram in the Space of the Analyzed GeneSet

  

Fig 3: KEGG\_HUNTINGTONS\_DISEASE: Random ES distribution      
 Gene set null distribution of ES for **KEGG\_HUNTINGTONS\_DISEASE**
